# Remote Control of Respiratory Neural Network by Spinal Locomotor Generators

**DOI:** 10.1371/journal.pone.0089670

**Published:** 2014-02-20

**Authors:** Jean-Patrick Le Gal, Laurent Juvin, Laura Cardoit, Muriel Thoby-Brisson, Didier Morin

**Affiliations:** Institut de Neurosciences Cognitives et Intégratives d'Aquitaine, CNRS, University of Bordeaux, Bordeaux, France; Emory University, United States of America

## Abstract

During exercise and locomotion, breathing rate rapidly increases to meet the suddenly enhanced oxygen demand. The extent to which direct central interactions between the spinal networks controlling locomotion and the brainstem networks controlling breathing are involved in this rhythm modulation remains unknown. Here, we show that in isolated neonatal rat brainstem-spinal cord preparations, the increase in respiratory rate observed during fictive locomotion is associated with an increase in the excitability of pre-inspiratory neurons of the parafacial respiratory group (pFRG/Pre-I). In addition, this locomotion-induced respiratory rhythm modulation is prevented both by bilateral lesion of the pFRG region and by blockade of neurokinin 1 receptors in the brainstem. Thus, our results assign pFRG/Pre-I neurons a new role as elements of a previously undescribed pathway involved in the functional interaction between respiratory and locomotor networks, an interaction that also involves a substance P-dependent modulating mechanism requiring the activation of neurokinin 1 receptors. This neurogenic mechanism may take an active part in the increased respiratory rhythmicity produced at the onset and during episodes of locomotion in mammals.

## Introduction

Upon initiation of exercise or locomotion, breathing rate rapidly increases in anticipation of the subsequent increased oxygen demand. Peripheral chemoreceptors provide one of the most important inputs for breathing regulation. However, chemoreception cannot account for this initial increase, because carbon dioxide and oxygen levels in the blood remain relatively constant at the onset of physical exertion [1], [2]. It is now widely recognized that the increase in respiratory rate observed at the onset of exercise, and sometimes even before exercise [3], [4], is mainly influenced by neural mechanisms [5], [6]. For instance, central commands originating either from the hypothalamic [7], [8], the mesencephalic [9], or the pontomedullary [10] locomotor region produce episodes of locomotion that are associated with a marked increase in respiratory activity. Thus, such feedforward mechanisms efficiently contribute to the increase in ventilation during exercise.

The rapid adjustment of breathing rate during exercise also involves muscular sensory feedback mechanisms. Known to be rhythmically activated during episodes of locomotion [11], [12], spinal peripheral afferents arising from limb muscle proprioceptors can promptly reset and entrain respiratory rhythmicity [13]–[18]. Indeed, cyclic activation of spinal sensory inputs is involved in the coupling between locomotor and respiratory rhythms in several species [19], and could also contribute to the exercise-induced increase in respiratory rate.

These feedforward and feedback mechanisms for functional interactions between respiratory and locomotor networks contribute to the functional coupling between breathing and locomotor behaviors. However, another attractive hypothesis would be to consider that direct influence from the locomotor central pattern generator (CPG) to the respiratory CPG would also constitute an optimal neurogenic mechanism, which would permit efficient and reliable adjustment of the respiratory CPG excitability. To test the existence of this previously unreported mechanism, we used an isolated *in vitro* brainstem-spinal cord preparation from neonatal rats [20]. This reduced preparation retains the capacity to generate both spontaneous respiratory rhythmicity originating from the medulla and pharmacologically induced locomotor rhythm produced in the lumbar spinal cord, thus allowing investigating potential direct interactions between the two central motor rhythm-generating networks. Here, we show that pharmacological activation of lumbar locomotor generator increases respiratory frequency. This effect requires the functional integrity of the parafacial respiratory group (pFRG) network and is mediated through a modulatory pathway dependent on substance P (SP). Furthermore, the increase in respiratory frequency observed during fictive locomotion is associated with a tonic depolarization of pre-inspiratory (Pre-I) neurons of the pFRG. Thus, our results assign the pFRG/Pre-I neurons a new role as relays in the pathway through which lumbar CPGs stimulate the medullary respiratory network during locomotion.

## Materials and Methods

### Newborn rat lines and care

All surgical and experimental procedures conformed to the guidelines of the European Communities Council Directive and the local ethics committee of the University of Bordeaux. The protocol was approved by the Committee on the Ethics of Animal Experiments of the University of Bordeaux (Permit Number: 5012031A). Experiments were performed on newborn Sprague Dawley rats (of either sex) that were obtained from females raised in our laboratory's breeding facility.

### *In vitro* isolated brainstem–spinal cord preparation

Neonatal rats (P0–2) were deeply anesthetized by hypothermia and decapitated. The skin and muscles were rapidly removed, and the preparation was then placed in a 25-ml recording chamber containing circulating artificial cerebrospinal fluid (aCSF) maintained at 10°C. The spinal cord with its dorsal and ventral roots still attached was isolated by dissection under a binocular microscope. The brainstem–spinal cord preparation was fixed on a Sylgard resin block with the ventral surface upward. Preparations were superfused continuously with aCSF (pH 7.4) equilibrated with 95% O_2_ and 5% CO_2_ and containing the following (concentrations in mM): NaCl 100, KCl 4, NaH_2_PO_4_ 1.2, CaCl_2_ 2, MgCl_2_ 1.3, NaHCO_3_ 25, and d-glucose 30. At the end of the dissection, the temperature of the bathing medium was progressively raised to 22–25°C before recordings began. This aCSF solution is commonly used to study the operation of locomotor rhythm-generating networks in isolated spinal cord preparation [21]–[24]. In these conditions, however, the elevated extracellular concentration of calcium (2 mM) induces a low baseline respiratory rate as observed in our experiments [25], [26]. Nevertheless, this reduced excitability is required to simultaneously record inspiratory- and expiratory-related motor activities for more than 2.5 hours (time necessary to achieve some of our protocols).

### Electrophysiological recording and stimulation

Motor output activity in spinal ventral roots and pFRG network bursting were recorded with glass suction electrodes filled with aCSF solution. Signals were amplified (10,000 times) by differential AC amplifiers (low cutoff, 100 Hz; high cutoff, 1 kHz; model 1700; A-M Systems), digitized and acquired via a CED 1401 interface, stored on a computer, and analyzed using Spike2 software (Cambridge Electronic Design).

Patch-clamp recordings of pFRG cells were made in whole-cell current clamp mode. Electrophysiological signals were recorded with an Axoclamp-2A amplifier (Molecular Devices), a digitizing interface (Digidata 1322A; Molecular Devices), and pCLAMP 10 software (Molecular Devices). Pipettes were pulled to a tip resistance of 5–7 MΩ and filled with solution containing (concentrations in mM) potassium gluconic acid 140, CaCl_2_·6H_2_O 1, EGTA 10, MgCl_2_ 2, Na_2_ATP 4, Hepes 10, and 0.1% biocytin. The pH was adjusted to 7.2.

Sacrocaudal dorsal roots were stimulated (30 pulses, 1 Hz, 0.5–1 V) via glass suction electrodes with a Master-8 simulator (A.M.P.I) controlled by software written in our laboratory.

### Drug application

Pharmacological substances were bath-applied at least 30 min after the end of the dissection. When required, the recording chamber was partitioned either into 2 or 3 compartments with barriers of syringe-ejected Vaseline to permit differential exposure of selected spinal cord regions to pharmacological treatment. The watertightness of the partitions was systematically checked at the end of the experiment by verifying that methylene blue added to one side of the Vaseline bridge did not leak into the other side. Experiments in which leakage was observed were discarded.

A mixture of *N*-methyl-d,l-aspartate (NMA; 15 µM; Sigma) and serotonin (5HT; 15 µM; Sigma) was applied to lumbar segments to elicit prolonged and stable episodes of fictive locomotion. In some cases, a sucrose solution (10%) or a modified aCSF solution in which the Ca^2+^ and Mg^2+^ concentrations were lower and higher, respectively, than those in the normal aCSF solution (0.1 mM CaCl_2_ and 5 mM MgCl_2_ *vs* 2 mM CaCl_2_ and 1.3 mM MgCl_2_) was bath-applied to the thoracic or cervical compartment to reversibly block axonal conduction or all chemical synaptic transmission [27], respectively.

The neurokinin 1 receptor (NK1R) antagonist spantide (Sigma) was applied to the brainstem region at a concentration of 5 µM. The preparation was pre-incubated in spantide for 1 h [28].

### Brainstem transection and lesion

Serial transverse sectioning of the brainstem was performed with a scalpel blade. To determine the involvement of brainstem structures in the locomotion-induced accelerated respiration, we transected the preparation rostral to the anterior inferior cerebellar artery (AICA) or we performed mechanical focal lesions of the brainstem with the tip of a glass suction electrode. The axial level of the transections (or lesions) was later anatomically verified on a parasagittal section of the brainstem.

### Histology

Following a transection or a lesion, the brainstem was stained for choline acetyltransferase. The tissue was fixed overnight at 4°C in 4% paraformaldehyde in PBS (0.1 M) and then rinsed twice in PBS and cryoprotected with 20% sucrose in PBS overnight. After the tissue was embedded in Tissue Tek and frozen to −80°C with cooled isopentane, 30-µm-thick serial parasagittal sections were cut on a cryostat. Free-floating sections were rinsed in PBS 3 times (each rinse lasted 10 min), saturated with PBS, Triton X100 0.3%, and BSA 1%, and incubated with a goat anti-choline acetyltransferase polyclonal antibody (1∶100, Millipore) for 18 h at room temperature (RT). Then the sections were rinsed in PBS, exposed to a donkey anti-goat IgG conjugated to Alexa Fluor 488 for 1 h at RT, and mounted in VECTASHIELD Hard-Set Mounting Medium (Vector).

The biocytin contained in the patch-clamp-recorded neurons was revealed with FITC 488–conjugated extravidin (1∶300; Sigma) in PBS solution for 1 h at RT. To investigate the expression of paired-like homeobox 2b (Phox2b) transcription factor, neurokinin 1 receptors (NK1R), and the motor neuronal marker Islet, we incubated the preparations with the following primary antibodies: a mouse anti-Phox2b antibody (1∶100; Santa Cruz), a rabbit anti-NK1R antibody (1∶10000; Sigma), and a mouse anti-Islet antibody (1∶250; DSHB). We then used goat anti-rabbit Alexa Fluor 568 or goat anti-mouse Alexa Fluor 488 secondary antibodies. The preparations were incubated with primary antibodies overnight under agitation at RT and with secondary antibodies for 1 h. Processed preparations were mounted in VECTASHIELD Hard-Set, and fluorescence was visualized with a Leica SP5 confocal microscope. The images were color corrected and assembled with the Leica LAS AF Lite viewer and Adobe Illustrator.

### Statistical analysis

Statistic analyses were carried out with SigmaPlot 11.0 (Systat), and results are expressed as mean ± SEM. Student's *t* test was used to compare the means of two groups, and repeated measures ANOVA and subsequent Tukey's post hoc tests were used to compare more than two groups. Differences were considered statistically significant when the value of *P* was <0.05.

## Results

### Pharmacological activation of lumbar CPG increased respiratory frequency

**Figure 1:**
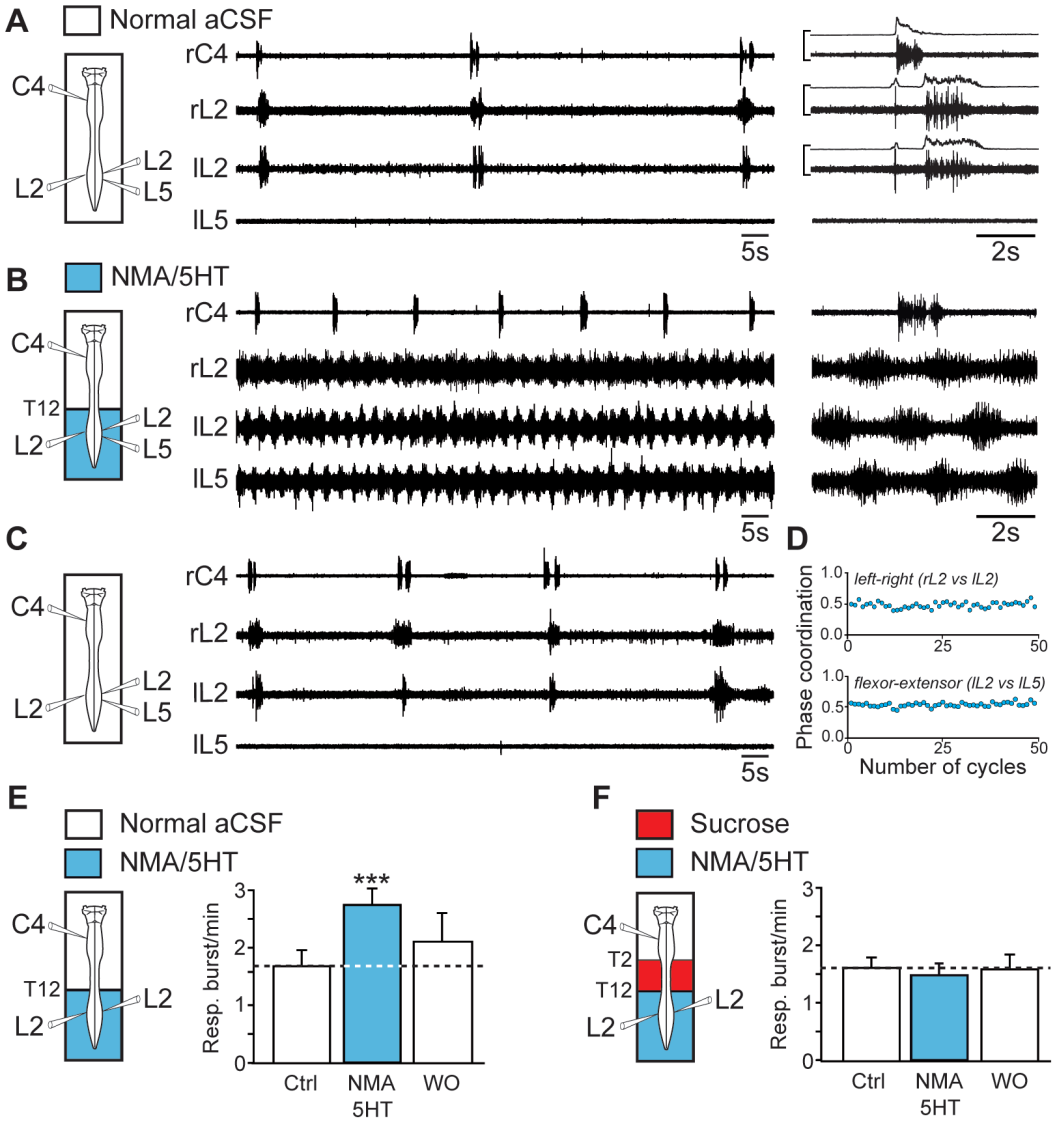
Activation of the hindlimb locomotor CPG increases the respiratory burst frequency. A, *left*, Schematic of the experimental procedure. *middle*, Raw spontaneous respiratory activity recorded from cervical (C4, inspiratory-like) and left (l) and right (r) lumbar (L2, expiratory-like) ventral roots. Note that under the control conditions, L5 motor output remained silent. *right*, Expanded traces of averaged integrated (9 sweeps) and raw activity recorded from the same ventral roots. B, Increase of the respiratory burst frequency during NMA/5HT-induced fictive locomotion (schematic at left). The layout is the same as that in (A). Expanded traces of the respiratory and locomotor bursts recorded from cervical (C4) and lumbar (L2 and L5) ventral roots, respectively, are shown at right. C, Recordings of respiratory activity during wash-out of NMA/5HT (schematic at left). D, Time course of the left–right (right L2 versus left L2, top) and flexor–extensor (left L2 versus left L5, bottom) phase coordination during fictive locomotion. Recordings and analysis shown in A to D are from the same preparation. E, F, *left*, Schematics of experimental setup. *right*, Bar charts showing the spontaneous respiratory burst frequency (mean ± SEM) under control (Ctrl) saline conditions (left, open bar), during NMA/5HT application to the lumbar region (blue bar), and during NMA/5HT wash-out (WO; right, open bar) when the lumbar segments of the cord were connected to the upper spinal and brainstem regions (E, n = 14 preparations) or disconnected by sucrose blockade of the thoracic (T2–T12) segments (F, n = 7 preparations). ***P<0.001 (repeated measures ANOVA, Tukey's post hoc tests).

In brainstem-spinal cord preparations, inspiratory- and expiratory-related motor activities recorded from cervical (C4) and lumbar (L2) ventral roots [29], [30], respectively, occurred at a low frequency (see Materials and Methods), with a mean value of 1.68±0.28 bursts/min in normal aCSF (n = 14 preparations; Fig. 1A and 1E, open bar at left). To elicit prolonged episodes of stable fictive locomotion, we bath-applied a mixture of NMA (15 µM) and 5HT (15 µM) selectively to the lumbosacral cord. In the presence of these neuromodulators, extracellular recordings from right and left L2 and left L5 ventral roots, which preferentially innervate hindlimb flexor and extensor muscles, respectively, showed coordinated rhythmic motor activity (Fig. 1B) characterized by strict left-right and flexor-extensor alternations (Fig. 1D). Strikingly, during these bouts of fictive locomotion, the respiratory burst frequency was 60% higher than the control value (2.74±0.28 bursts/min; *P*<0.001; n = 14 preparations; Fig. 1B and 1E, blue bar). It is noteworthy that in preparations showing a higher baseline respiratory rate (5 bursts/min), we previously reported that pharmacological activation of the lumbar cord still produced a significant 30% respiratory rhythm acceleration [16]. In the present study, this increase was reversible after 30 min of wash out (Fig. 1C and 1E, open bars at right) and was no longer observed when axonal conduction in the thoracic spinal segments (T2–T12) was prevented by means of sucrose blockade (Fig. 1F). These findings strongly suggest that, when active, the hindlimb locomotor CPG exerted an excitatory ascending influence on the medullary respiratory rhythm generators.

**Figure 2:**
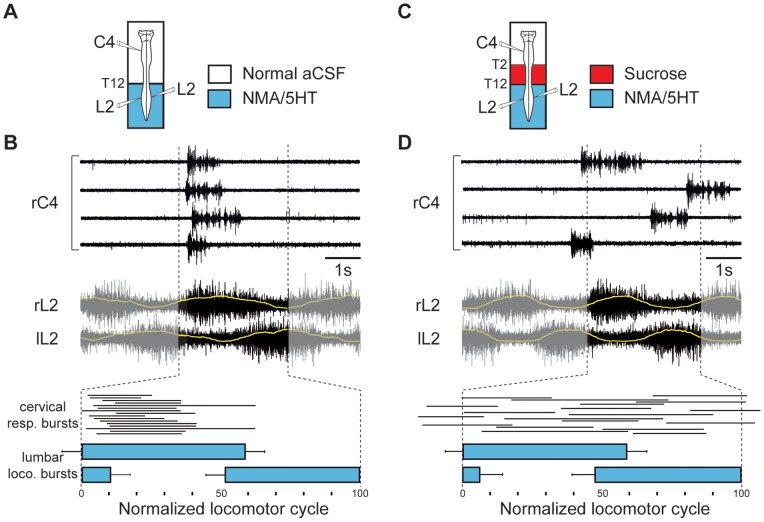
Phase relationship between respiratory and locomotor bursts. A, C, Schematics of experimental setup. B, D, *upper and middle traces*, Raw respiratory and locomotor activity recorded from right cervical (rC4) and left-right lumbar (rL2 and lL2) ventral roots, respectively, when lumbar spinal region was connected to the upper spinal and brainstem structures (B) or disconnected by a sucrose blockade of thoracic segments (D). Yellow lines superimposed on rL2 and lL2 discharges are an averaging (30 successive locomotor cycles) of integrated locomotor ventral root activity. *lower diagrams*, Phase diagrams illustrating normalized locomotor step cycles during pharmacologically evoked fictive locomotion. Note that cervical respiratory (resp.) bursts (thin horizontal lines) occur preferentially in-phase with lumbar locomotor (loco.) discharges (blue horizontal bars) when hindlimb locomotor CPG are connected to the medullary respiratory generators (B). Recordings and analysis shown in B and D are from the same preparation.

During locomotion, lumbar CPG rhythmically produces time-dependent excitation phases at the origin of locomotor bursting. We assumed, therefore, that the caudorostral influence from the lumbar locomotor network to the respiratory centers should be maximal during the generation of a locomotor burst activity. Indeed, during episodes of stable fictive locomotion, a clear locomotor-respiratory temporal relationship occurred, which consisted of an in-phase relationship between C4 respiratory and ipsilateral L2 locomotor bursts (Fig. 2A and 2B). The respiratory burst onset invariably occurred during the first 20% of the locomotor cycle and shortly after lumbar locomotor burst onset (mean delay 370±47 ms; n = 57 motor bursts from 8 preparations; Fig. 2B). Finally, this in-phase relationship was no longer observed when lumbar locomotor CPG and medullary respiratory generators were disconnected by sucrose blockade of thoracic (T2–T12) spinal segments (n = 8; Fig. 2C and 2D). These data show that the central modulation of respiratory center burst activity by locomotor networks can contribute to the coupling between breathing and locomotor rhythms.

### Sacrocaudal afferent stimulation-induced fictive locomotion caused increased respiratory rhythmicity

**Figure 3:**
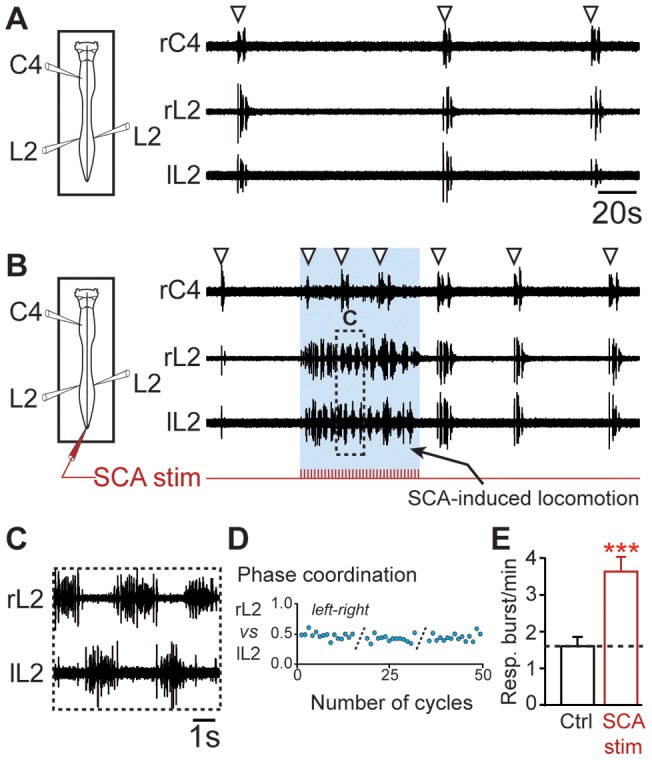
Electrical stimulation of SCA activates the lumbar locomotor CPG and increases the respiratory rate. A, *left*, Schematic of experimental procedure. *right*, Spontaneous respiratory activity from cervical (C4) and right (r) and left (l) lumbar (L2) ventral roots. White arrowheads denote detected respiratory bursts. B, Fictive locomotion and increase in respiratory burst frequency induced during the tonic stimulation of SCA. The layout is the same as that in (A). Expanded traces of fictive locomotor burst activity recorded from bilateral L2 ventral roots in (B). D, Plot showing left-right locomotor phase coordination occurring between bilateral flexor (L2) ventral roots during 3 successive episodes of SCA stimulation–induced locomotion. E, Bar charts illustrating changes in respiratory burst frequency (Resp.; mean ± SEM; n = 7 preparations) following SCA stimulation. Recordings and analysis shown in A to D are from the same preparation. Ctrl, control. *** *P*<0.001 (Student's paired *t* test).

Tonic electrical stimulation of sacrocaudal afferents (SCA) is an alternative, drug-free method of activating lumbar locomotor CPG [31]–[33]. SCA stimulation also induced a rhythmic locomotor-like activity recorded in left and right L2 ventral roots (Fig. 3B and 3C), characterized by bilateral burst alternation (n = 7 preparations; Fig. 3D). Similar to the effect observed after pharmacological activation of the lumbar cord, a significant increase in respiratory burst frequency occurred during SCA stimulation (1.61±0.23 bursts/min under control conditions vs 3.62±0.36 bursts/min during SCA stimulation; P<0.001; n = 7 preparations; Fig. 3A, 3B and 3E) and could extend for several tens of seconds after the end of stimulation (Fig. 3B). These findings further support the conclusion that the activation of locomotor CPG in the lumbar region can modulate the activity of respiratory rhythm generators.

### Pathways mediating locomotion-induced modulation of respiratory rhythm

**Figure 4:**
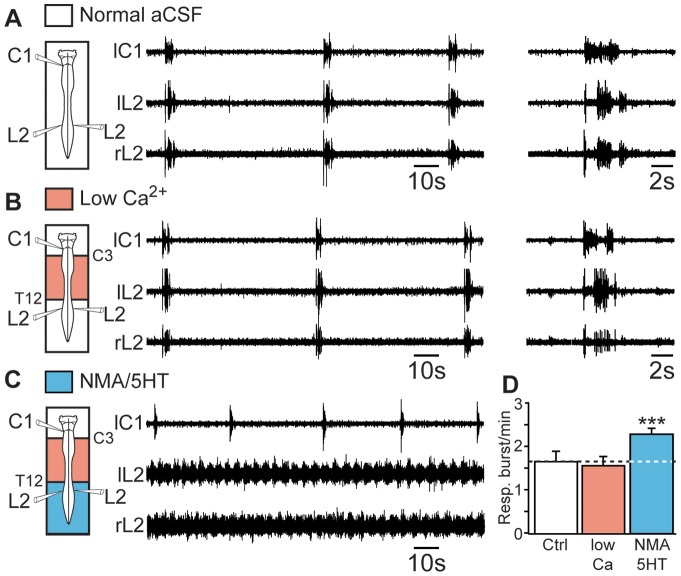
Excitatory influence of lumbar locomotor networks on respiratory centers does not require supralumbar spinal synaptic relays. A, B, C, *left*, Schematics of experimental setup. *middle*, Raw spontaneous respiratory activity recorded from cervical (C1, inspiratory-like) and lumbar (L2, expiratory-like) ventral roots under control conditions (A), during the application of a low-Ca^2+^/high-Mg^2+^ saline solution (Low Ca^2+^) to the cervicothoracic (C3–T12) segments (B), and during subsequent activation of the lumbar locomotor CPG with a mixture of NMA and 5HT (C). Note that the respiratory pattern remained unchanged after the pharmacological blockade of cervicothoracic synaptic transmission (expanded traces at right in A and B) with the low-Ca^2+^/high-Mg^2+^ saline solution. D, Bar charts illustrating changes in respiratory burst frequency (Resp.) under these 3 experimental conditions (mean ± SEM; n = 8 preparations). Recordings shown in A to C are from the same preparation. Ctrl, control conditions. ***P<0.001 (repeated measures ANOVA, Tukey's post hoc tests).

In the context of quadrupedal locomotion, four-limb coordination relies on a powerful caudorostral synaptic influence that originates from the lumbar locomotor CPG and reaches the cervical cord level through activation of intervening thoracic segments [34]. To determine whether the increase in respiratory burst frequency evoked during lumbar fictive locomotion was also mediated by synaptically relayed ascending pathways, we blocked chemical synaptic transmission in thoracic and cervical segments with a low-Ca^2+^/high-Mg^2+^ medium and simultaneously activated the lumbosacral cord with a combination of NMA and 5HT. First, the blockade of chemical synaptic transmission in spinal segments extending from C3 to T12 altered neither the frequency of the ongoing respiratory rhythm (1.62±0.25 bursts/min under control conditions *vs* 1.53±0.22 bursts/min under blockade conditions; n = 8 preparations; Fig. 4A, 4B and 4D) nor the pattern of inspiratory and expiratory phases recorded from cervical (C1) and lumbar (L2) ventral roots, respectively (Fig. 4A and 4B, expanded traces on right). Second, when fictive locomotion was subsequently pharmacologically induced in the lumbar cord region, the respiratory rhythm frequency was significantly increased (2.25±0.14 bursts/min; *P*<0.001; n = 8 preparations; Fig. 4C and 4D). We therefore propose that the influence of the hindlimb locomotor CPG on the respiratory centers relies on nonrelayed ascending supralumbar spinal pathways.

### Pontine structures are not involved in the acceleration of respiration induced by activation of lumbar locomotor CPG

**Figure 5:**
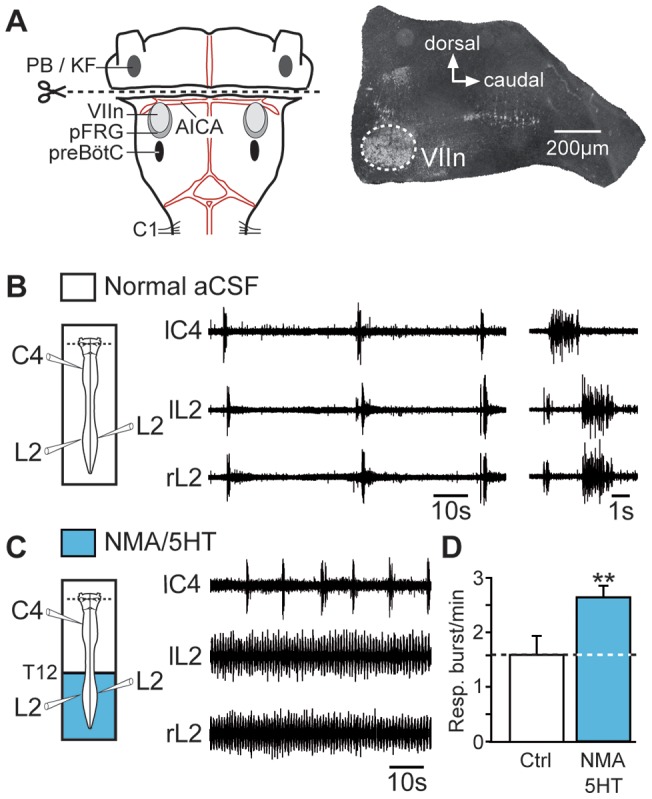
Pontine structures are not engaged in the accelerated respiration induced by activation of the lumbar locomotor CPG. A, *left*, Schematic drawing showing the level of transection (dotted line) performed rostral to the AICA. Note that this transection allowed removal of pontine structures of the brainstem while preserving medullary nuclei. *right*, Histological control of the brainstem transection. The parasagittal section, which was immunostained for acetylcholinesterase, clearly shows the transection rostral to the facial nucleus (VIIn). B, C, *left*, Schematic of experimental procedures. *middle*, Raw spontaneous respiratory activity recorded from cervical (C4, inspiratory-like) and left (l) and right (r) lumbar (L2, expiratory-like) ventral roots under control conditions (B, with expanded traces shown at right) and during the pharmacological activation of the lumbar locomotor generators with NMA/5HT (C). D, Bar charts showing changes in respiratory burst frequency (Resp.; mean ± SEM; n = 7 preparations) under control saline conditions (open bar) and during NMA/5HT application to the lumbar spinal region (blue bar). Ctrl, control conditions; PB/KF, parabrachial/Kölliker–Fuse complex. **P<0.01 (Student's paired *t* test).

In an attempt to identify supraspinal structures engaged in the modulation of respiratory rhythmicity, a series of transections was made just rostral to the AICA to remove pontine structures (n = 7 preparations; Fig. 5A), including the parabrachial/Kölliker-Fuse complex, which is known to play a crucial role in locomotor-respiratory couplings [17], [18]. In this medullary preparation, a normal respiratory-related activity pattern was maintained (Fig. 5B), showing inspiratory and expiratory phases monitored from C4 and L2 ventral roots, respectively (Fig. 5B, expanded traces on right). We also observed that the pharmacological induction of locomotor-like activity in the lumbar cord networks significantly increased the frequency of spontaneous respiratory rhythm (1.58±0.34 bursts/min under control conditions *vs* 2.62±0.21 bursts/min under pharmacological induction; *P*<0.01; n = 7 preparations; Fig. 5C and 5D), as previously observed in pontomedullary preparations. These results demonstrate that pontine structures are not required for the modulation of respiratory rhythm by the lumbar locomotor CPG.

### Lumbar modulation of respiratory rhythm required functional parafacial respiratory groups

**Figure 6:**
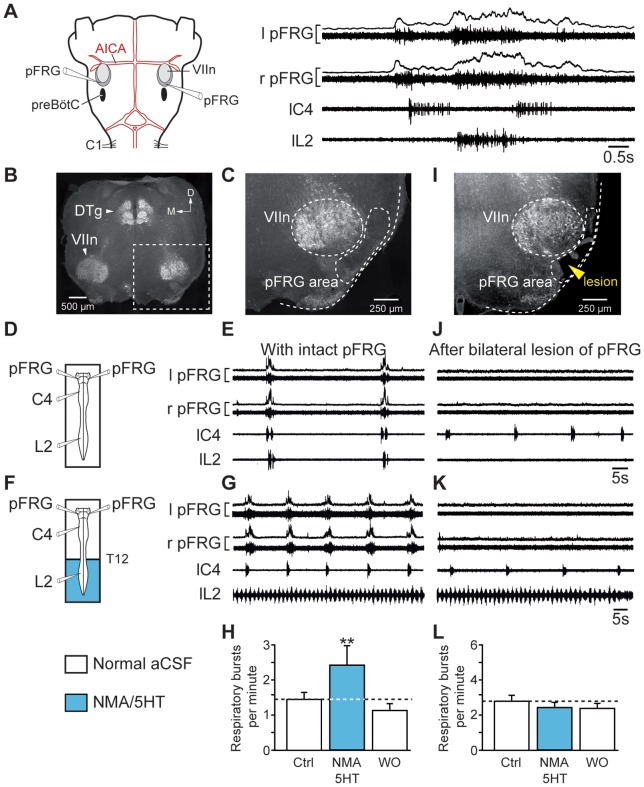
Bilateral lesion of the pFRG suppresses the locomotion-induced accelerated respiration. A, *left*, Representation of the brainstem–spinal cord preparation with medullary recording sites. *right*, Averaged integrated (6 sweeps) and raw respiratory burst activity recorded from the left (l) and right (r) pFRG and from spinal (cervical C4 and lumbar L2) ventral roots. B, C, I, Transverse acetylcholinesterase-stained sections as histological control in intact (B, C) and lesioned (I) brainstem preparations. The position of the lesion ventral to the facial motor nucleus corresponds to the location of the pFRG (I). D, F, Schematics of experimental setup. E, J, Spontaneous respiratory activity recorded bilaterally from the pFRG and homolaterally from cervical (C4) and lumbar (L2) ventral roots under control conditions in intact preparation (E) and after the bilateral lesion of the pFRG (J). G, K, Respiratory effects of lumbar cord activation with NMA/5HT in intact (G) and pFRG-lesioned (K) brainstem-spinal cord preparations. H, L, Bar charts showing variation in the respiratory burst frequency (Resp.; mean ± SEM) under control conditions (open bar, left), during pharmacological activation of lumbar locomotor CPG (blue bar), and after wash-out (open bar, right) in intact (H, n = 7 preparations) and pFRG-lesioned (L, n = 7 preparations) preparations. Recordings shown in E, G, J and K are from the same preparation. VIIn, facial motor nucleus; Ctrl, control conditions; DTg, dorsal tegmental nucleus; D, dorsal; M, medial; WO, wash-out. **P<0.01 (repeated measures ANOVA, Tukey's post hoc tests).

The above finding supports the conclusion that ascending influence from lumbar locomotor CPG has access to medullary structures engaged in the modulation of respiratory rhythmic activity. It is now widely admitted that respiratory rhythm is generated by two distinct but functionally interacting rhyhtmogenic oscillators, the parafacial respiratory group (pFRG) and the pre-Bötzinger complex (preBötC; for review see [[35]). Because we previously reported that pFRG-driven lumbar expiratory activity was first evoked during respiratory entrainment by limb proprioceptive afferents in the neonate [18], we investigated here the potential involvement of pFRG in the caudorostral influence of locomotor CPG on central respiratory networks. We conducted experiments combining direct electrophysiological recording of pFRG activity and subsequent mechanical bilateral lesions of the pFRG region. Extracellular recording performed in the pFRG region showed pre- and post-inspiratory discharges (Fig. 6A) occurring in a typical double bursting pattern [29], [36] responsible for the respiratory lumbar nerve activity [25], [29], [30]. With intact (Fig. 6B and 6C) and functionally active pFRG network (Fig. 6E), activation of lumbar locomotor generators (Fig. 6F and 6G) resulted in a significant increase in respiratory burst frequency (1.44±0.21 bursts/min under control conditions *vs* 2.44±0.46 bursts/min; *P*<0.01; n = 7 preparations; Fig. 6H). After the bilateral lesion of pFRG network, which was corroborated by the loss of bursting in L2 ventral root recordings (Fig. 6J) and verified histologically (Fig. 6I), the ability of hindlimb locomotor CPG to modulate the central respiratory burst frequency was abolished (n = 7; Fig. 6K and 6L). Taken together, these results provide evidence that in the neonatal rat, neural components of the pFRG network are involved in the upward influence exerted by the lumbar CPG on respiratory centers during episodes of fictive locomotion.

### Cellular and neuromodulatory mechanisms for locomotor-respiratory network coordination

**Figure 7:**
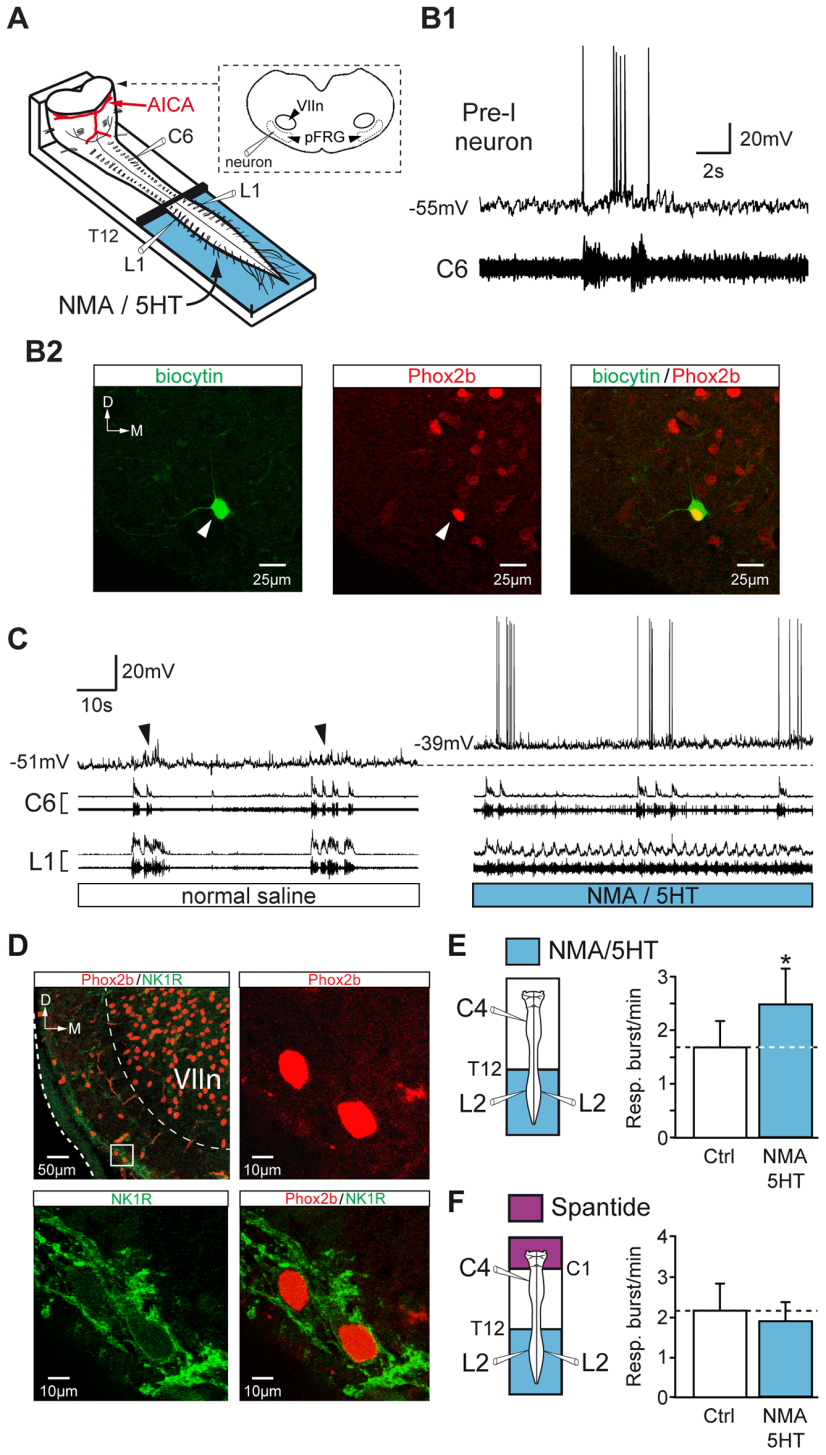
Depolarization of pFRG neurons during pharmacologically induced locomotion in the lumbar spinal cord: involvement of an SP pathway. A, Schematic representation of the preparation. The brainstem was transected rostrally to the AICA to allow direct access to pFRG neurons for patch-clamp recording. B1, Simultaneous whole-cell patch-clamp recording of a pFRG/Pre-I neuron and raw activity of the cervical (C6, inspiratory-like) ventral root under control conditions. B2, Photomicrographs (z-stack of 3 images, 1.8 µm in total thickness) of immunolabeling for Phox2B (middle, red) in a biocytin-filled neuron (left, green). The white arrowhead indicates the neuron recorded in B1, and the merged image (right) confirms that the double-labeled (yellow) cell was a functionally identified Pre-I neuron. The dashed line shows the ventrolateral edge of the medulla. C, Simultaneous whole-cell patch-clamp recording of a pFRG/Pre-I neuron (black arrowhead indicating spontaneous rhythmic respiratory depolarization) and integrated and raw activity of cervical (C6) and lumbar (L1) ventral roots under control saline conditions (left) and during NMA/5HT application to lumbar region (right). The dashed line indicates the resting membrane potential level under control conditions. D, Left, top, Distribution of NK1R (green) immunoreactivity and Phox2B-positive cells (red) in the ventrolateral part of the medulla (this photomicrograph corresponds to a z-stack of 18 images, 18 µm in total thickness). The inset (white rectangle) shows the ventrolateral aspect of the VIIn at a higher magnification. Note that cells located in the area corresponding to the pFRG networks are immunopositive for Phox2b (right, top, red; z-stack of 3 images, 2.4 µm in total thickness) and for NK1R (left, bottom, green), as confirmed by the merged image (right, bottom). E, F, *left*, Schematics of experimental procedures. *right*, Bar charts showing variation in the respiratory burst frequency (Resp.; mean ± SEM) under control saline conditions (open bar) and during NMA/5HT application to the lumbar spinal cord (blue bar) during perfusion of the brainstem with normal saline (E, n = 7 preparations) or with a medium containing the SP antagonist spantide (F, n = 7 preparations). Ctrl, control conditions; D, dorsal; M, medial. *P<0.05 (Student's paired *t* test).

We next examined the cellular mechanisms underlying the locomotion-induced increase in respiratory rate. Using a preparation (adapted from [36]) giving direct access to the parafacial region at the cut surface of the transection (Fig. 7A), whole-cell patch-clamp recordings were performed from pFRG neurons (n = 10 cells) in current-clamp mode. Located ventral to the facial motor nucleus and close to the ventral surface of the brainstem, pFRG/Pre-I neurons displayed spontaneous periodic membrane depolarizations characterized by the presence of pre- and post-inspiratory discharges (Fig. 7B1) and were immunoreactive for the transcription factor Phox2b (n = 6/6 cells; Fig. 7B2), as previously described [37], [38]. Activation of lumbar locomotor networks by NMA/5HT-containing saline induced a tonic depolarization of pFRG/Pre-I neurons by 6.1±1.3 mV (n = 10 neurons; Fig. 7C). This membrane depolarization, which was partially reversible (3.2±0.7 mV; n = 6 neurons), was associated with an increase in action potential firing, indicating a general increase of pFRG/Pre-I neuron excitability during drug-evoked fictive locomotion in the lumbar spinal cord.

Previous studies have shown that Phox2b-immunoreactive cells in the parafacial region also express NK1R [38]–[40] and that pFRG/Pre-I neurons are depolarized by the NK1R agonist SP [41]. Therefore, in a final series of experiments, we investigated the possibility that a release of SP in the medulla was involved in the respiratory burst frequency acceleration that results from the activation of lumbar locomotor CPG. First, NK1R was expressed in the area where patch-clamp recordings were performed, ventrally to the facial motor nucleus (Fig. 7D, upper left microphotograph). Moreover, we confirmed that Phox2b-immunoreactive neurons of the pFRG also expressed NK1R (Fig. 7D). Second, we investigated whether the respiratory burst frequency modulation produced by locomotor CPG was mediated by a SP-releasing pathway. The significant increase in respiratory burst frequency evoked during fictive locomotion in response to NMA/5HT activation of lumbar cord (1.70±0.49 bursts/min under control conditions *vs* 2.47±0.67 bursts/min during fictive locomotion; *P*<0.05; n = 7 preparations; Fig. 7E) was no longer observed when the brainstem was selectively exposed to the NK1R antagonist spantide (5 µM; n = 7 preparations; Fig. 7F). These results support the conclusion that the ascending influence of the lumbar locomotor CPG activates the release of SP, which in turn leads to the acceleration of the breathing rate.

## Discussion

The results described in this report provide evidence indicating that lumbar locomotor pattern generators can participate to the modulation of respiratory network activity during episodes of locomotion. The modulation is mediated by ascending excitatory information that is conveyed to central respiratory networks through an SP-releasing pathway within the brainstem. During activation of hindlimb locomotor CPGs, pFRG/Pre-I neurons are tonically depolarized and show a global excitability increase that could be part of neurogenic mechanisms responsible for accelerating breathing rate during locomotion. Our findings suggest that in addition to playing a role in respiratory rhythm generation and central chemoreception [35], [41], [42], pFRG/Pre-I neurons also appear to be engaged in the interaction between locomotor and respiratory neural networks.

### A spinal efference copy signal to modulate respiratory network activity

It is now widely recognized that spinal locomotor networks are continuously controlled by several brainstem centers and descending pathways [43]–[45] that in turn receive ascending feedback information arising from both peripheral sensory systems and spinal locomotor CPGs [46]. Specifically, this CPG-derived efference copy, which is part of a spino-bulbo-spinal loop in the lamprey [47] and spinocerebellar tracts in the cat [48], strongly contributes to rhythmic activity in reticulospinal [49]–[51] and vestibulospinal neurons [52], thereby actively participating in the adaptation of the locomotor movements and posture during locomotion. At the spinal level, this locomotor-related efference signal also appears to be essential for coordinating back and limb muscle contractions during appendicular swimming or quadrupedal walking. For instance in *Xenopus* froglets, postural adjustments are mediated by projections that ascend from the hindlimb lumbar CPG to the thoracic spinal circuitry controlling the trunk musculature [53]. Similarly, in neonatal rats, lumbar locomotor CPG-related commands have been found to coordinate trunk curvature with hindlimb stepping [54] and also to contribute to interlimb coordination during locomotion by means of coupling between the lumbar pattern generator and its cervical counterpart [24], [34].

It is noteworthy that during locomotion, these ascending copies of lumbar output can also assist distant neural networks engaged in different rhythmic motor behaviors. Indeed, ascending efference copies of rhythmic activities produced by the locomotor CPG within the lumbar cord of *Xenopus* tadpoles [55], [56] and frogs [57] have recently been reported to target the brainstem extraocular motor nuclei to help stabilize gaze during undulatory and appendicular swimming, respectively. Our results show that CPG-derived efference copies can also be engaged in the remote control of respiratory rhythm-generating networks. Compatible with feedforward mechanisms to rapidly accelerate the rate of respiratory movements at the onset of exercise [9], the ascending spinal influence could also participate to the emergence of a locomotor-respiratory coupling. Indeed, we report here that an in-phase relationship occurs between locomotor and respiratory burst activity, a phenomenon that could assist the locomotor-respiratory coupling generated by the rhythmic activation of limb proprioceptive afferents during locomotion [16], [18]. Finally, we demonstrate that the transmission of the lumbar CPG-derived influence to the respiratory centers is mediated mainly by direct spinal ascending nerve tracts, rather than by polysynaptic pathways. A similar anatomical organization, involving a direct spinal ascending pathway, has also been reported for gaze stabilization during locomotion in *Xenopus* [56], [57]. In these studies, the drive signals from the spinal locomotor CPG are directly targeted to extraocular motoneurons during swimming. We hypothesize that this direct connectivity promotes rapid, efficient coordination between two otherwise functionally and anatomically distinct motor systems.

### pFRG/Pre-I neurons: new “motion sensors” in locomotor-respiratory coordination?

The last decade of research on central respiratory circuits has provided several lines of evidence showing that the pFRG contributes to the rhythmogenesis of respiration during the perinatal period in rodents [36], [38], [58], [59], contributes to the production of active expiration [25], [30], [60], [61], and plays a major role in central chemoreception [40], [62]–[68]. Here, our lesion and patch-clamp experiments demonstrate for the first time that the pFRG cell population is also implicated in the locomotor-respiratory interaction. In this context, pFRG/Pre-I neurons may convert the lumbar CPG-derived efference copy into excitatory signals, which would be subsequently transmitted to the whole respiratory network. Indeed, the functional connections that exist between the pFRG and other crucial respiratory nuclei [69], [70] allow the widespread transmission of these excitatory inputs in other neuronal groups involved in the genesis or the modulation of respiratory rhythm. Interestingly, this functional organization closely resembles the one implicated in central mechanisms for chemosensitivity. Indeed, the increase in respiratory rate observed during acidosis [71] is likely due to the excitation of pFRG neurons [40], [62], [67] because *in vitro*, the preBötC, known to be responsible for generating inspiration phase [72], is not intrinsically sensitive to pH changes [65].

In our experiments, the acceleration of respiratory rhythm during locomotion was suppressed when the brainstem was initially exposed to spantide, an NK1R antagonist. As previously reported in rodents [38]–[40] and in humans [73], we show that Phox2b-positive neurons in the pFRG region also express NK1R. Therefore, SP that is co-localized with serotonin in terminals located in the pFRG [39] and is known to excite pFRG/Pre-I neurons [41], [67], [74] is suspected to play a role in pFRG/Pre-I cell depolarization induced by the lumbar CPG during locomotion. Although its precise contribution to the process of coordination remains undetermined, this SP-dependent link would contribute to the transformation of a phasic ascending input from the lumbar locomotor CPG into a tonic excitatory signal. Consequently, the long-lasting depolarization of pFRG/Pre-I neurons, which activates their burst-generating mechanisms, could therefore underlie the rapid adjustment of breathing rate that occurs at the onset of exercise.

Does ascending influence from the lumbar locomotor CPG stimulate the preBötC? An action of SP on the preBötC neurons during locomotion cannot be completely ruled out. Indeed, NK1R is strongly expressed in the preBötC region [75]–[78], and exposure of the preBötC network to SP evokes in inspiratory neurons a membrane depolarization [75], [79] or a voltage-insensitive inward current [80] leading to an increase in the respiratory frequency [28], [75], [79], [81]. But after bilateral lesions that removed the pFRG and left intact the preBötC, we demonstrated that the ability of lumbar CPG to modulate the respiratory rhythmicity was clearly abolished. These results tend to suggest that the pFRG would be the main oscillator directly engaged in the lumbar locomotor CPG-induced increase in respiratory rate. In any case, further experiments are required to fully elucidate the exact role of preBötC neurons in the functional interaction between the locomotor and respiratory CPGs.
